# Piloting a long-term evaluation of library data workshops

**DOI:** 10.5195/jmla.2021.1047

**Published:** 2021-07-01

**Authors:** Fred Willie Zametkin LaPolla, Nicole Contaxis, Alisa Surkis

**Affiliations:** 1 fred.lapolla@med.nyu.edu, Knowledge Management Librarian at the NYU Health Sciences Library, Data Services team, liaison to General Internal Medicine and Radiology, NYU Health Sciences Library, New York, NY; 2 nicole.contaxis@nyulangone.org, NYU Health Sciences Library, New York, NY; 3 alisa.surkis@nyulangone.org, NYU Health Sciences Library, New York, NY

**Keywords:** library evaluation, data education, data science, library workshops

## Abstract

**Background:**

Over four years of hosting library data workshops, we conducted post-workshop evaluation of attendees' satisfaction with the workshops but not longer-term follow-up. To best allocate library resources and most effectively serve the needs of our users, we sought to determine whether our data workshops were impactful and useful to our community. This paper describes a pilot project to evaluate the impact of data workshops at our academic health sciences library.

**Case Presentation:**

We surveyed individuals who signed up for data workshops between 2016 and 2019. Surveys included open-ended and multiple-choice questions, with the goal of having participants describe their motivations for taking the workshop(s) and how they ultimately used what they learned. An analysis of responses using the Applied Thematic Analysis model indicated that the workshops had an impact on the respondents, although the strength of our conclusions is limited by a relatively low response rate.

**Conclusions:**

Survey results indicated that our workshops impacted how researchers at our medical center collect and analyze data, supporting the conclusion that we should concentrate our educational efforts on providing skills-based workshops. The low response rate and time-consuming nature of the analysis point toward several improvements for future evaluation efforts, including better tracking of workshop attendees, a shorter survey with fewer open-ended questions, and survey implementation within one year of the workshop date.

## BACKGROUND

The past decade has witnessed growth in the provision of data services by academic health sciences libraries, with new service models including training in research data management, data analysis, and coding for data science in languages like Python and R [1–10]. In our academic health sciences library, we began offering data services in the form of research data management workshops in 2012. Beginning in 2016, our library started regularly hosting a series of data workshops that included instruction by library faculty and staff, as well as guest instructors from across the medical center and our broader university community [5]. By the term “data workshops,” we mean stand-alone educational sessions that include topics broadly related to research data, including data management, data collection, data visualization, and data analysis. After each workshop, we conducted attitudinal surveys of participant experience. We also instituted regular biannual retreats for library faculty and staff to engage in reflective practice [1], but we had not initially done longer-term follow-up with workshop participants.

Educational programming in libraries is not new [11], but data-focused education represents a shift in the medical library landscape [12]. Because data services are a relatively new area of medical library work, we found relatively little literature focused on evaluation of data education programming. Coates et al. [1] describe the use of reflective practice on the part of librarian-instructors in evaluating workshops, and Deardorff [13] used a mixed-methods study to evaluate the impact of Carpentries workshops on participants. There are also case studies of data education workshops that included descriptions of attitudinal evaluations [1, 4, 5, 8, 14] wherein users are asked to provide immediate feedback on workshops after they are completed. Skills-based evaluation, where participants demonstrate what they have learned, are less common, although Partridge et al. described having participants demonstrate their learning by making shareable graphics in Google's suite of office tools [9].

In looking beyond the library literature to the field of professional development and adult education, we found additional guidance. Kutner et al. provide a description of different elements of education that might be evaluated, such as the ability of instructors, institutional support, and change in learners, and techniques for evaluation [15]. Guskey notes that there remains little consensus on what criteria should be evaluated and that many would-be assessors are frustrated by the complexity of the issue [16]. Indeed, Knowles [17] notes that assessing the impact of education on behavior is a very fraught activity given the complexity of systems being examined and the many elements that can lead to change in a person's behavior.

This case report details our efforts to conduct long-term follow-up with data workshop participants to improve the workshops and determine how best to use limited resources. This paper aims to fill a gap in the literature by providing an example of a project evaluation in the medical library data services sphere.

## CASE PRESENTATION

### Survey implementation and analysis

To gather information on the impact of our data workshops, we conducted an online survey of workshop attendees. As we were not able to identify any validated instruments for our purposes, we formulated our own research instrument, reaching consensus among authors on the content and structure of the survey that would elicit useful information without overwhelming potential respondents. Our survey consisted of multiple-choice questions, with an option for “other,” regarding respondents' role, department, school/hospital affiliation, and workshops attended followed by open-ended questions (Appendix 1). The open-ended questions allowed respondents to explain their experiences [18] and provide information on why they took workshops, what impact the workshops had on their work life, how they used the material, and what new topics were of interest. This study was approved by the New York University Langone Health (NYU Langone) Institutional Review Board (i19001694).

The survey was disseminated using a listserv consisting of individuals who signed up for our data workshops between 2016 and 2019. Not all individuals on the listserv necessarily attended workshops, as the list included those who cancelled registrations, signed up but did not attend, or were on waitlists. We used this as a proxy for attendees because we had not maintained a comprehensive database of attendees. Our survey was emailed to 1,278 individuals, but 230 email addresses were undeliverable, leaving a pool of 1,048 individuals. Two reminder emails were sent.

The analysis followed the Applied Thematic Analysis method [18]. The three authors reviewed the open-ended text responses and generated codes iteratively to identify themes. After we reached consensus on codes, each written response was coded by two authors using Google Docs spreadsheets, which was downloaded as a Microsoft Excel file to convert and upload into R Studio as CSVs. We analyzed coded responses and information on participation affiliation, role, and workshops taken with descriptive statistics using R Studio version 1.2.1335 with R version 3.6.1 (2019-07-05).

### Respondent demographics

Of the 1,048 individuals to whom the survey was delivered, 108 opened and 60 completed the survey, making the response rate 5.7% (60/1,048). Of the 60 respondents who completed the survey, the majority were affiliated with the School of Medicine or Health System (e.g., clinical staff, project staff on clinical research teams), and the rest were from the College of Nursing, College of Dentistry, or Graduate Biomedical program (Table 1). Internally available data from evaluations conducted immediately after the workshops showed that 85% of attendees were affiliated with the School of Medicine or Health System, mirroring the present results.

**Table 1:** Affiliations of survey respondents

Affiliation	Number of survey respondents	Percentage of survey respondents	Percentage of workshop attendees
NYU Grossman School of Medicine	36	60.0	67.6
NYU Langone Health	15	25.0	18.2
Rory Myers College of Nursing	6	10.0	4.9
NYU College of Dentistry	1	1.7	3.0
Graduate Biomedical Institute	1	1.7	1.4
Other	1	1.7	4.9

Respondents were divided among a number of different roles, with no single role dominating. Nearly a quarter of respondents were faculty, and under a fifth were research/project coordinators. No other group represented more than 10% of respondents (Table 2). Compared to all workshop participants, faculty were overrepresented among the survey respondents (23.33% of survey respondents versus 13.22% of all workshop participants).

**Table 2:** Roles of survey respondents

Role	Number of survey respondents	Percentage of survey respondents	Percentage of workshop attendees
Faculty	14	23.3	13.2
Project/research coordinator	11	18.3	22.4
Postdoc	6	10.0	13.1
Project/program/research manager	6	10.0	4.6
Data analyst	5	8.3	5.1
Administrator	4	6.7	4.2
Graduate student	3	5.0	5.5
Medical student	1	1.7	4.3
Intern	1	1.7	4.4
Other	9	15.0	22.5

### Workshops attended and self-reported learning

The sixty respondents reported attending a total of 167 workshops (Table 3). Of these respondents, nearly three-quarters attended a REDCap workshop, over one-quarter attended an R workshop, and one in five attended a research data management workshop or a data visualization class (not including a workshop on the R package ggplot2, which was categorized as an R workshop).

**Table 3:** Workshops taken by survey respondents

Workshop title	Percentage of respondents who attended (respondents could attend more than one workshop)
Getting Started with REDCap	53.3
Introduction to R	36.7
Designing Longitudinal Studies and Surveys in REDCap	35.0
Advanced REDCap	26.7
Clinical Research Data Management	20.0
Data Visualization Clinic	15.0
Research Data Management Essentials	15.0
Other	15.0
Data Visualization with Excel	13.3
Data Science for Non-Data Scientists	10.0
Data Visualization with GraphPad Prism	10.0
Data Visualization with ggplot2	10.0
Improving Data Collection Workflows in REDCap	8.3
Data Transfer at NYU Langone	5.0
Data Visualization Best Practices	1.7
Introduction to Git and GitHub	1.7
Statistical Process Control for Quality Improvement	1.7

While the survey was sent to all individuals who signed up for a workshop within the last four years, 66% of respondents had attended a workshop in 2019. As only 23% of all individuals emailed signed up for a workshop in 2019, this subset is over-represented.

Fifty-nine respondents reported how frequently they used what they learned in the workshops through a multiple-choice question; 8% (n=5) said every day, 24% (n=14) said every week, 27% (n=16) said every month, 34% (n=20) said a few times a year, and 7% (n=4) said they never used what they learned.

Sixty respondents indicated how they used what they learned through another multiple-choice question; 63% (n=38) indicated using what they learned to do their jobs more quickly, 45% (n=27) indicated they used what they learned to think differently about challenges, 23% (n=14) highlighted using materials to approach challenges in a new way, and 5% (n=3) indicated they used what they learned in some other way.

### Respondents' motivations for workshop attendance

Forty-three respondents described their motivations for attending a workshop(s) through an open-ended question. Many respondents (42.5%, n=17) indicated that they wanted to build a skill, for example: “The reports generated from REDCap for our database are not at all user friendly. I was trying to understand how I could improve upon them while I work with IT to come up with a better way to analyze data[,]” and “It facilitated familiarity with tools I've never worked with but knew of. Instead of self-teaching myself when a project was due it was better to learn when it wasn't needed professionally so I can explore how to use the new program in my day to day versus just to accomplish one project.”

Other respondents (35%, n=14) indicated that they wanted to gather information on a topic in a general manner without specifically stating it was for a skill-related goal, for example: “To determine whether it was worthwhile to have staff take these courses. In other words, whether these tools would be valuable to our work.”

Some respondents (25%, n=10) indicated wanting to learn a skill in order to use it in a current role, which was determined by an explicit reference to their use in work obligations, for example: “I knew that in my new role I would be using REDCap more and I wanted to be able to independently use the program, rather than relying on colleagues to assist or answer all my questions. I achieved this through taking the workshop.”

Additionally, one respondent (2%) indicated using a skill for a change in career, and one other respondent (2%) indicated they took a workshop for the benefit of an in-person learning experience.

### Respondents' use of what they learned

Forty-four respondents described how they used what they learned in the workshop(s) through an open-ended question. Half of respondents (51%, n=19) indicated using what they learned in the data collection process, for example: “Introduced me to RedCap and slicer/dicer, both tools I use regularly now. Made me aware of resources available to me.”

Some respondents (28%, n=11) indicated using what they learned for data analysis, for example: “Yes, now that my lab has begun doing single-cell RNA-Seq, it is much easier to understand code that we are given or use and to modify it for our own needs.” Other respondents indicated using what they learned in their current position (21%, n=8); to create scholarly products like papers, posters; or figures (15%, n=6), or educating others on their team (8%, n=3).

### Impact on respondents' work life

Forty-five respondents described the impact the workshop had on their work life, which was broadly defined to mean any change to their work or way of thinking about problems, in an open-ended question. Many respondents (32%, n=13) described a change to their professional workflow, for example: “Using RedCap helps to streamline our data and allows participants to access our assessments from anywhere.” Many other respondents (27%, n=11) described being introduced to a new concept (27%, n=11), for example, “the R class gave me the necessary foundation to begin learning how to do more advanced coding on my own.” Some respondents indicated that they gained a new professional skill (15%, n=6) or deepened or refreshed a skill that they already possessed (15%, n=6), as exemplified by “The class I took was a great refresher course for me. I had been working in research for about five years and wanted to make sure that I was still doing things by the book.” Other respondents described gaining confidence to instruct others (e.g., colleagues) in a skill learned (12%, n=5) or to approach a task (10%, n=4), for example: “Primarily it has reduced my anxiety about using the different tools presented to me and has expanded my tool box.” A few respondents indicated gaining a new perspective on a topic (7%, n=3), thinking of the library as a hub for data education (7%, n=3), or seeing no impact (7%, n=3).

### Respondents' interest in new topics

Finally, 36 respondents described their interest in topics not already covered by the workshops in an open-ended question. Respondents indicated interest in more REDCap training (30%, n=7), more R programming (26%, n=6), training in project management (26%, n=6), more data analysis training (13%, n=3), more data management training (5.5%, n=2), and training in Excel (2.8%, n=1) and Python (2.8%, n=1).

## DISCUSSION

While we only succeeded in gathering responses from a small percentage of those we surveyed, their responses indicated that our workshops had an impact, particularly in introducing attendees to new concepts and providing insight into topics that can be used in their professional lives. Many individuals attended workshops to build skills and gather information and used what they learned for data collection and analysis. This makes intuitive sense to us, given that most respondents attended REDCap and R workshops, which are data collection and data analysis tools, respectively. Furthermore, most respondents indicated a desire for more training in R programming and REDCap.

Because this study focuses on local workshop conditions, the results are not generalizable in other contexts. Moreover, as Knowles [17] notes, many elements outside of the classroom could influence whether individuals put their learning to use in their broader professional lives. For example, a person might have professional responsibilities that cause them to do more or less data collection regardless of whether they took a workshop on data collection.

We encountered numerous challenges in conducting a long-term programmatic evaluation of the impact of our data workshops. A core issue was the low response rate of 5.7%, which partly reflects a major problem with our recruitment methodology, in that we emailed everyone who had ever signed up for a workshop rather than limiting to those we knew had attended. As a result, our list likely included many people who never attended a workshop. Additionally, the length of time that had elapsed since the workshops may have depressed turnout. While we surveyed attendees of workshops offered between 2016 and 2019, two-thirds of respondents attended a workshop in 2019. Moreover, faculty were overrepresented in our survey sample, possibly reflecting the lower turnover of this role. By contrast, research staff tend to have much higher turnover and, along with students, may be the main contributors to the 18% (230/1,278) of undeliverable email addresses.

While our intent in providing open-ended questions was to give space for respondents to describe their experiences, the reality is that often responses presented us with limited actionable information and greatly expanded the time needed to analyze data compared to multiple-choice questions. An alternate approach would be to offer more multiple-choice questions, which would aid in expediting analysis and provide suggestions to users about possible new topics or areas of impact that they might not have considered. For example, many of the requests for new topics in workshops reflected areas we already teach, but providing a curated list of data topics in which other libraries provide instruction might broaden the scope of answers.

Due to the limitations discussed, this pilot evaluation provided limited actionable information but allowed some insights about our respondents' needs. We learned that most respondents are primarily motivated by information-seeking needs as opposed to general interest, leading us to infer that we should focus primarily on goal-oriented learning experiences rather than, for example, networking tasks or broad general interest talks [17].

This pilot will serve as a building block for future evaluation work, in which we are taking two approaches to improving our response rate. First, we updated our data collection workflows to maintain a record of attendance. Second, given that the majority of survey respondents had taken a workshop in the last year, we will now conduct programmatic evaluation on a yearly basis while experiences are fresh. Our approach to eliciting more actionable information from respondents will be to provide more multiple choice questions, which could help broaden the range of answers. As offering multiple choice questions runs the risk of creating “leading” questions that provide a limited or distorted picture of user experiences, development of valid and reliable evaluations requires an iterative process that balances concerns of overly leading participants with the limitations of free-text responses. An additional advantage of multiple choice questions is that they save time in analyzing user responses by allowing for quantitative analysis, which is an important consideration in ensuring the sustainability of conducting an annual workshop evaluation.

## Data Availability

The survey instrument, anonymized data, and codebook are available at https://figshare.com/authors/Fred_LaPolla/8980472.

## SUPPLEMENTAL FILES

**Appendix 1** Survey instrument

## References

[1] Coates H, Carlson J, Clement R, Henderson M, Johnston L, Shorish Y. How are we measuring up? Evaluating research data services in academic libraries. J Librarianship Scholarly Communication. 20182018;6(General Issue). DOI: 10.7710/2162-3309.2226.

[2] Brandenburg MD, Garcia-Milian R. Interinstitutional collaboration for end-user bioinformatics training: cytoscape as a case study. J Med Libr Assoc. 2017 4;105(2):179–84. https://www.ncbi.nlm.nih.gov/pmc/articles/PMC5370611/pdf/jmla-105-179.pdf. 28377683 10.5195/jmla.2017.224 PMC5370611

[3] Federer L. Beyond the Basics: Pushing the Limits of Data Management Education. Austen, TX: Medical Library Association; 2015.

[4] Federer LM. Beyond data management: developing a comprehensive data science support program in the library. Toronto, Canada: Medical Library Association; 2016.

[5] Surkis A, LaPolla FW, Contaxis N, Read KB. Data day to day: building a community of expertise to address data skills gaps in an academic medical center. J Med Libr Assoc. 2017 4;105(2):185–91. https://www.ncbi.nlm.nih.gov/pmc/articles/PMC5370612/pdf/jmla-105-185.pdf. 28377684 10.5195/jmla.2017.35 PMC5370612

[6] Deardorff A. Why do biomedical researchers learn to program? An exploratory investigation. J Med Libr Assoc. 2020 1;108(1):29–35. https://www.ncbi.nlm.nih.gov/pmc/articles/PMC6920002/pdf/jmla-108-29.pdf. 31897049 10.5195/jmla.2020.819 PMC6920002

[7] LaPolla FWZ, Rubin D. The “Data Visualization Clinic”: a library-led critique workshop for data visualization. J Med Libr Assoc. 2018 10;106(4):477–82. https://www.ncbi.nlm.nih.gov/pmc/articles/PMC6148617/pdf/jmla-106-477.pdf. 30271289 10.5195/jmla.2018.333 PMC6148617

[8] Read K, LaPolla FWZ. A new hat for librarians: providing REDCap support to establish the library as a central data hub. J Med Libr Assoc. 2018 1;106(1):120–6. https://www.ncbi.nlm.nih.gov/pmc/articles/PMC5764577/pdf/jmla-106-120.pdf. 29339942 10.5195/jmla.2018.327 PMC5764577

[9] Partridge E, Vaidhyanathan V, Hoover B, Gotschall T, Beard L, editors. Establishing a successful data visualization service: lessons from the field. Chicago, IL: Medical Library Association; 2019.

[10] Foster E, Champieux R. Hosting a science hack day…and you can too! Seattle, WA: Medical Library Association; 2017.

[11] Weill S. Transformed from a cemetery of bric-a-brac. In: Shepperd B, editor. Perspectives on outcome based evaluation of libraries and museums. Washington, DC: Institute of Museum and Library Services; 2000.

[12] Cox AM, Kennan MA, Lyon L, Pinfield S. Developments in research data management in academic libraries: towards an understanding of research data service maturity. J Assoc Inf Sci Technol 2017;68(9):2182–200. DOI: 10.1002/asi.23781.

[13] Deardorff A. Assessing the impact of introductory programming workshops on the computational reproducibility of biomedical workflows. PLoS One 2020;15(7):e0230697. 32639955 10.1371/journal.pone.0230697 PMC7343163

[14] Ragon B. Where is my data scientist?Austin, TX: Medical Library Association; 2015.

[15] Kutner M, Sherman R, Tibbetts J, Condelli L. Evaluating professional development: a framework for adult education. Washington, DC: Pelavin Research Associates; 1997.

[16] Guskey TR. What makes professional development effective? Phi Delta Kappan. 2003;84(10):748–50. DOI: 10.1177/003172170308401007.

[17] Knowles MS, Holton EF III, Swanson RA. The adult learner: the definitive classic in adult education and human resource development. 6th edition. London, UK: Taylor & Francis; 2005.

[18] Guest G, MacQueen K, Namey EE. Applied thematic analysis. Thousand Oaks, CA: Sage Publishing; 2012. Available from: https://methods.sagepub.com/book/applied-thematic-analysis.

[19] Nitecki DA. Program evaluation in libraries: relating operations and clients. Arch Sci 2004;4(1-2):17–44. http://link.springer.com/10.1007/s10502-005-6989-3.

[20] Nitecki DA, Hernon P. Measuring service quality at Yale University's libraries. J Acad Librariansh 2000;26(4):259. DOI: 10.1016/S0099-1333(00)00117-8.

