# Supplementary material for: Piloting a long-term evaluation of library data workshops
Source: J Med Libr Assoc. 2021 Jul 1;109(3):490–6. doi: 10.5195/jmla.2021.1047 (PMC8485943; doi:10.5195/jmla.2021.1047)
Supplement: Supplementary file 1 — Appendix 1 Survey instrument [file jmla-109-3-490-s01.pdf]

Data Day To Day Opinionnaire

You are being invited to take part in a research study entitled Library Evaluation Analysis Project for Reviewing Observable Gains.

Purpose: The purpose of this research study is to better understand the impact of Health Sciences Library data workshops on the work of members of the NYU Langone community with the hope of improving our future workshops.

Key Information and Study Procedures: This study will last from December 2019 to May 2020 and has two parts. For the first part, you will be asked to take part in an online survey about your opinions of the impact that participating in a library data workshop has had on your work-life. We anticipate it will take approximately 10 minutes to fill out. The second part of the study consists of a 1-hour semi-structured follow up interview to help us better understand your views on the impact of the workshops and what parts of classes you found to be most meaningful. At the end of the online survey, you will be given an option to indicate your willingness to participate in the second part.

Risks: There are minimal risks for taking part in this study. There is a possibility that some of the questions may make you uncomfortable or distressed. You don't have to answer those questions if you don't want to. If you decide to participate in the second part of our study, the follow-up interview, we will collect your name and email but will store this information only on MCIT secured drives and only share them with study personnel. We will only report de-identified information. Your responses will not be shared with your supervisor or anyone else who is not on the study team, and participation will have no impact on your employment, salary or performance review.

Benefits: There are no direct benefits to you. Your participation may help us improve future educational workshops.

Confidentiality: All participant information is completely confidential and identifying information will not be shared with non-study personnel. We are storing your information in REDCap, an MCIT secured, HIPAA-compliant tool and will only report de-identified information. We are collecting name and email so that if you are willing to engage in an in-person interview we are able to contact you.

Alternatives: Participation in this study is completely voluntary. You can choose to not to take part in this study. Decision whether or not to participate in this study will have no impact on your employment, salary, or performance evaluation.

Cost and Compensation: There are no cost or compensation to you for taking part in this research study.

Contact: If you have questions about the study please contact the PI:

Fred LaPolla, MLS
212.263.8535
fred.lapolla@nyulangone.org

If you have questions, concerns or complaints regarding your participation in this research study or if you have any questions about your rights as a research subject, you should speak with the Principal Investigator. If a member of the research team cannot be reached or you want to talk to someone other than those working on the study, you may contact the Institutional Review Board (IRB) at (212) 263-4110.

Do you consent to participate in this study?

◯ Yes
◯ No

Library Evaluation Analysis Project for Reviewing Observable Gains

Affiliation
- ◯ School of Medicine
- ◯ College of Dentistry
- ◯ College of Nursing
- ◯ NYU Langone Health
- ◯ Sackler
- ◯ Other

If other affiliation, please specify ______________________

Department/Division ______________________

Role
- ◯ Faculty
- ◯ Postdoc
- ◯ Medical Student
- ◯ Graduate Student
- ◯ Resident
- ◯ Administrator
- ◯ Intern
- ◯ Project/Research Coordinator
- ◯ Project/Program/Research Manager
- ◯ Data Analyst
- ◯ Other

If other role, please specify ______________________

Which workshop(s) did you attend at the Health Sciences Library, check all that apply

☐ Clinical Research Data Management ☐ Data Science for Non-Data Scientists ☐ Data Visualization Clinic ☐ Data Visualization with Excel ☐ Data Visualization with GraphPad Prism ☐ Data Visualization Best Practices ☐ Data Visualization with ggplot2 ☐ Designing Longitudinal Studies and Surveys in REDCap ☐ Improving Data Collection Workflows in REDCap ☐ Getting Started with REDCap ☐ Advanced REDCap ☐ Introduction to R ☐ Introduction to Git and GitHub ☐ Research Data Management Essentials ☐ Reproducibility Workshop ☐ Statistical Process Control for Quality Improvement ☐ Latent Variables and Factor Analysis ☐ Data Transfer at NYU Langone ☐ Other

When did you last attend a workshop at the Health Sciences Library?
- ◯ Summer 2016
- ◯ Fall 2016
- ◯ Spring 2017
- ◯ Summer 2017
- ◯ Fall 2017
- ◯ Summer 2018
- ◯ Spring 2019
- ◯ Summer 2019

How has attending a library workshop impacted your work or life if at all? By impact we mean has allowed you to work, think, research or act in a way that you otherwise might not have. ______________________

How often have you used what you learned in the workshop? This can include in concrete ways (such as using a tool) or in abstract ways (such as ideas informing your work or approach to work).
- ◯ Never
- ◯ A few times a year
- ◯ Every month
- ◯ Every week
- ◯ Every day

How have you used what you learned in the workshop? This can include in concrete ways (such as using a tool) or in abstract ways (such as ideas informing your work or approach to work).	__________________________________
How have you used what you learned in the workshop? This can include in concrete ways (such as using a tool) or in abstract ways (such as ideas informing your work or approach to work).	☐ To accomplish tasks related to my job more effectively or efficiently<br>☐ To approach challenges in my job in a new way<br>☐ To think differently about optimal ways to accomplish a task<br>☐ Other
Why did you decide to take the workshop that you attended? By this question we mean, what purpose did the learning serve for you, or what made you feel the need to take a workshop	__________________________________
Based on what you now know, are there new things you would like to learn or topics that were not covered that you would have appreciated learning about? What are they?	__________________________________
Thinking about your experience, what would you improve?	__________________________________
Thinking about your experience, what would you want kept the same?	__________________________________
Would you be willing to be involved in an in-person interview to discuss your experience further?	◯ Yes<br>◯ No
Name:	____________________________
Email:	____________________________

Thank you for your time, please hit submit to submit your responses.
